# Demystifying visual awareness: Peripheral encoding plus limited decision complexity resolve the paradox of rich visual experience and curious perceptual failures

**DOI:** 10.3758/s13414-019-01968-1

**Published:** 2020-01-22

**Authors:** Ruth Rosenholtz

**Affiliations:** MIT Department of Brain & Cognitive Sciences, CSAIL, Cambridge, MA 02139 USA

**Keywords:** Peripheral vision, Attention, Decision complexity, Awareness, Search, Change blindness, Scene perception

## Abstract

Human beings subjectively experience a rich visual percept. However, when behavioral experiments probe the details of that percept, observers perform poorly, suggesting that vision is impoverished. What can explain this awareness puzzle? Is the rich percept a mere illusion? How does vision work as well as it does? This paper argues for two important pieces of the solution. First, peripheral vision encodes its inputs using a scheme that preserves a great deal of useful information, while losing the information necessary to perform certain tasks. The tasks rendered difficult by the peripheral encoding include many of those used to probe the details of visual experience. Second, many tasks used to probe attentional and working memory limits are, arguably, inherently difficult, and poor performance on these tasks may indicate limits on decision complexity. Two assumptions are critical to making sense of this hypothesis: (1) All visual perception, conscious or not, results from performing some visual task; and (2) all visual tasks face the same limit on decision complexity. Together, peripheral encoding plus decision complexity can explain a wide variety of phenomena, including vision’s marvelous successes, its quirky failures, and our rich subjective impression of the visual world.

## 1. Introduction

At any given moment, the human visual system clearly faces limits, both in terms of the information available and the tasks one can successfully perform. Because of these limits, real-world vision involves an iterative process. We start with some—possibly unconscious—task (i.e., some question about the world). For instance, we might start by asking, “What is the layout of this room?” We do our best to complete that task. If necessary, we can gain more information by taking actions such as moving our eyes. In the next instance, we shift to another task to gain more understanding of the visual world. For example, we might next query, “Are there any people here?” Similarly, the contents of our awareness of the visual world shift from moment to moment.

When we attempt to characterize our understanding and awareness of the visual world, a fundamental puzzle arises. On one hand, we subjectively experience a rich visual world, effortlessly perceived (Dennett, 1991; Noë, 2002). However, when probed on the details, observers know surprisingly little (as reviewed in the next paragraph). The rich experience suggests a highly capable visual system, whereas poor performance when reporting details suggests that perception is impoverished. For the purposes of this paper, I refer to this puzzling combination of rich subjective experience and poor objective task performance as the *awareness puzzle*—though it is far from the only puzzle when it comes to understanding awareness (Tononi, Boly, Massimini, & Koch, 2016).

For example, we subjectively experience real-world scenes as rich and detailed (Dennett, 1991). However, change a portion of that scene while masking transients that would provide a cue, and observers have difficulty noticing what changed (e.g., Rensink, O’Regan, & Clark, 1997). Similarly, while we experience a rich percept of an ensemble of similar items, observers perform poorly when asked to report the features of a particular item (Ariely, 2001; Chong & Treisman, 2005; Haberman & Whitney, 2009). Furthermore, it is often difficult to search for a particular target item unless it has a distinct basic feature such as orientation, color, or motion (Wolfe & Horowitz, 2004). Search can be difficult even when, upon examination, target and distractors appear quite distinct (e.g., when searching for a “T” among “L”s). Difficult search, then, suggests that the details that distinguish the search items must be unavailable; otherwise, search would be easy.

An influential theory—feature integration theory, or FIT (Treisman & Gelade, 1980)—proposed that poor search performance arises from a particular kind of limited capacity: limited access to higher level processing. According to this theory, observers can quickly and easily perform tasks that require only basic feature maps; such tasks rely only on *preattentive* visual processing. However, any tasks that rely on binding or conjoining an object’s features, such as distinguishing a “T” from an “L,” require *selective attention*. According to this theory, attention serially selects what information travels through the limited capacity channel to receive higher level processing.

**Fig. 1:**
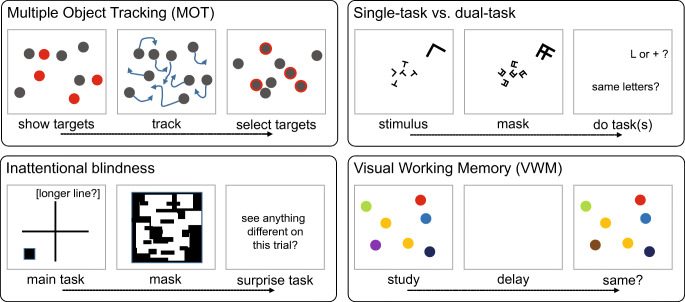
Visual search, change detection, and perception of individual items of a set suggest that perception is limited without attention. Meanwhile, paradigms such as multiple object tracking, dual-task, and inattentional blindness have suggested that attention is limited. Visual working memory tasks, in turn, have suggested that memory has limited capacity. In each paradigm depicted, time advances to the right, as indicated by the arrow. This paper argues that these tasks are inherently difficult.

Attention, in turn, appears to have greatly limited capacity (see Fig. 1). Multiple object tracking (MOT) tasks, for instance, have been taken to suggest that observers can attend to and track only about four objects at a time (e.g., Pylyshyn & Storm, 1988; although for another view of MOT, see Franconeri, Alvarez, & Cavanagh, 2013). Furthermore, there is often a cost to performing more than one task at once (e.g., VanRullen, Reddy, & Koch, 2004), particularly when one of the tasks is unknown to the observer, as in the phenomenon of inattentional blindness (Mack & Rock, 1998).

If perception is poor without attention, and attention has limited capacity, then at a given instant, we cannot perceive very much. Furthermore, we cannot merely build up a rich percept by rapidly shifting attention and remembering what we have previously perceived because visual working memory itself appears to have a low capacity of approximately four items (e.g., Luck & Vogel, 1997). While some researchers have questioned this item limit account in favor of more flexible resources (e.g., Fougnie, Cormiea, Kanabar, & Alvarez, 2016; Ma, Husain, & Bays, 2014; Palmer, 1990), this theoretical difference does not obviously allow one to use memory to build a richer percept. Arguably to make full use of a flexible memory resource one first needs richer perception, perhaps through a flexible attentional resource (e.g., Treisman, 2006).

This description of the awareness puzzle focuses heavily on somewhat old-fashioned theories of attention and working memory. Throughout this paper, I will refer to these theories of selective attention (e.g., Treisman & Gelade, 1980; Wolfe, Cave, & Franzel, 1989), multiple object tracking (e.g., Pylyshyn & Storm, 1988), and visual working memory (e.g., Luck & Vogel, 1997) as the *classic* theories. Considerable work has gone into updating these theories, particularly in the case of attention (Carrasco, 2011). The classic theories may as a result appear to be straw men. However, they are important because they lead to the awareness puzzle. With the exception of a few theories developed to address this puzzle (described next), the updated theories do not obviously solve it.

Some philosophers and vision researchers have noted the confusing collection of phenomenology described above and have proposed theories to address the underlying puzzles. The first two theories are philosophical in nature and attempt to make sense of the apparent contradiction between the rich subjective experience and poor performance at a number of objective tasks. The second set of theories, more vision science than philosophy, suggest mechanisms to account for both the awareness puzzle and for real-world vision.

The first philosophical theory, here referred to as the illusion theory, suggests that the rich subjective impression is merely an illusion and is therefore not incompatible with the impoverished perception observed in behavioral experiments (Blackmore, Brelstaff, Nelson, & Troscianko, 1995; Dennett, 1991, 1998; O’Regan, 1992; Rensink, O’Regan, & Clark, 1997).^1^ This theory must contend with empirical evidence in favor of an objectively richer percept. Observers can rapidly get the gist of a scene (e.g., Greene & Oliva, 2009; Loschky et al., 2007; Loftus & Ginn, 1984; Potter, 1975; Potter & Fox, 2009; Rousselet, Joubert, & Fabre-Thorpe, 2005), and this gist includes rich information about that scene (Fei-Fei, Iyer, Koch, & Perona, 2007). Similarly, we can rapidly extract properties of an ensemble (Alvarez, 2011; Ariely, 2001; Chong & Treisman, 2003, 2005; Haberman & Whitney, 2009). Clearly, these results are, at minimum, problematic for the original FIT, as noted in Treisman (2006), although it remains unclear whether the details objectively available to observers suffice to explain the subjective experience.

The second philosophical theory posits that we are aware of more than we can act upon (Block, 2011; Lamme, 2010). In this theory, here referred to as the inaccessibility theory, the rich percept is real, but the information is perversely inaccessible when it comes to making decisions or otherwise taking action. At face value, this proposal seems counterintuitive. Visual awareness is likely *more* limited than perception, not less. Organisms can carry out considerable visual processing without awareness (Helmholtz, 1867; Koch & Crick, 2001).

It is not obvious how either philosophical theory would lead to a working visual system. If perceptual richness is mere illusion, how are we so successful at so many visual tasks? As for the inaccessibility theory, generating a rich percept requires significant processing on the part of the visual system; why would an organism put energy and effort into awareness, but not ensure the ability to act on the available information?

Vision science theories have attempted to account for the awareness puzzle while also explaining how real-world vision might work. One class of theories, for instance, focuses on the question of how, if preattentive vision is so poor, and attention so limited, we can intelligently shift attention to gather more information. How can we reasonably form and test new hypotheses to gain understanding about the visual world? Suppose I want my coffee mug. To identify it, I need to attend to it; where do I direct my attention? Knowing the gist and/or the layout of the scene would help, but in the early, classic theories of attention, it was not obvious how to get that information from either the preattentive feature maps or limited selective attention. It might help me to know that the mug sits on the desk. However, this presents a chicken-and-egg problem: I would have to attend to the desk to identify the desk. If it is my desk, in my office, I might have prior knowledge of its location. If it is someone else’s desk, but I know it is brown, I could use crude preattentive features to filter for brown stuff (Wolfe, Cave, & Franzel, 1989). What if I know neither piece of information? For that matter, how do I ever perceive task-irrelevant parts of the scene, such as a person sitting at the desk?

Mack and Rock (1998), noting that their inattentional blindness studies seemed to suggest little or no perception without attention, proposed that some information must be capable of *capturing* attention. They reviewed attempts to uncover the rules of attentional capture. Stimulus-driven, or bottom-up, capture could occur if the information is sufficiently *salient* (Theeuwes, 1992), though this might depend upon the task set (Folk, Remington, & Johnston, 1992). Bottom-up saliency (i.e., unusual features) could be computed from the hypothesized preattentive features (e.g., Itti & Koch, 2001; Rosenholtz, 1999). Capture by salient items could help us notice interesting parts of the scene even if they are not task relevant. Top-down filters could also reveal task-irrelevant information. For instance, Simons and Chabris (1999) noted that observers more frequently notice an unexpected gorilla walking through a basketball game when counting passes of the team wearing black jerseys. They suggested that the filter for “black” might accidentally capture the gorilla, leading to identification (the capture is accidental, even though the filter selected black as intended, because the goal is to select teammates with black jerseys, not gorillas). However, taking a step back, attentional capture seems like an odd proposal for how vision might work: The visual system makes up for poor preattentive processing both by being easily distracted by irrelevant salient stuff and by having top-down filters accidentally capture task-irrelevant items with crude low-level similarity to the task-relevant items. This is no way to design a visual system, and it seems unlikely that capture can explain vision’s successes (Nakayama, 1990; Rosenholtz, Huang, & Ehinger, 2012). (Mack and Rock (1998) themselves instead came to support late selection rather than attentional capture theory.)

A second class of vision theories has built on the observation that classic selective attention theory can account for some of vision’s quirky failures (hard search, change blindness, and inattentional blindness, to name a few), but is problematic when it comes to explaining vision’s marvelous successes. This might suggest that the visual system augments the selective attention pathway with additional information. Scenes and sets, for example, might be processed in a separate, nonselective pathway (Cohen, Dennett, & Kanwisher, 2016; Rensink, 2000; Wolfe, Vo, Evans, & Greene, 2011). Alternatively, different modes of attention might make available different information; diffusely attend to a scene as a whole and get the gist, or attend to a set of items and gain access to ensemble properties like the mean size (Nakayama, 1990; Treisman, 2006). In these latter theories, the system switches between different attentional modes, as opposed to having separate pathways running simultaneously. Both theories assume that the additional mechanisms (separate pathway or different attentional modes) use a different sort of encoding, unlike that for ordinary object recognition (Cohen, Dennett, & Kanwisher, 2016; Nakayama, 1990; Treisman, 2006; Wolfe, Vo, Evans, & Greene, 2011). Researchers have suggested that mechanisms might encode some sort of summary statistics that would support both scene and ensemble tasks (Cohen et al., 2016; Haberman & Whitney, 2011; Oliva & Torralba, 2006; Treisman, 2006; Wolfe et al., 2011). Similarly, Rensink (2000) describes an underlying representation not of basic feature maps, but rather in terms of more complex *proto-objects*, resulting from low-level computation of local geometric and photometric properties. In his theory, summary statistics of proto-objects support computation of gist and layout of the scene.

This paper does not argue that these vision science theories cannot solve the awareness puzzle, but rather that we can do better, based first on a modern understanding of peripheral vision. The second section of this paper, “An Efficient Encoding in Peripheral Vision Explains Many of the Puzzles of Vision,” reviews a concrete, testable hypothesis for the encoding in peripheral vision, and argues that this encoding can explain performance on several of the tasks critical to the awareness puzzle. However, this is not to say that these phenomena encounter no additional limits. Important as peripheral vision is, it cannot completely solve the awareness puzzle on its own. However, by attributing as much as we can to peripheral vision, we gain a clearer idea of what phenomenology remain unexplained. In the third section, “A Proposal for an Additional Capacity Limit: Limited Decision Complexity,” I suggest that these remaining phenomenology follow a pattern. Based on this pattern, I hypothesize that an additional capacity limit on decision complexity will account for many of the remaining phenomena. In the fourth section, “Additional Comparison With Existing Theories,” I discuss advantages of the proposed two-part hypothesis. Of course, visual processing has additional mechanisms not discussed here; the goal is to identify a minimal set of general-purpose limits that, once understood, make sense of a wide range of seemingly unrelated visual phenomena: our rich subjective experience, the limited detail we can report, and the power of real-world vision. If we can do this, then we can consider ourselves to have made sense of the awareness puzzle.

This paper largely avoids the term *attention* throughout the second and third sections. I do this for clarity, as the term has an overloaded definition that means different things to different people and in different contexts. The paper discusses pointing one’s eyes at an object without reference to overt attention, and it similarly discusses the *task* of monitoring a subset of display items—for example, in response to a cue—without raising the issue of an attentional *mechanism* for performing that task. Most critically, I initially present the hypothesized limit on decision complexity without reference to attention, to avoid confusion with attention’s myriad definitions. That said, some readers may wish to make connections between the theory described in this paper and various concepts of attention. I will later draw some of these parallels and suggest advantages for reframing attention in terms of limited decision complexity.

## An efficient encoding in peripheral vision explains many of the puzzles of vision

### Change blindness and difficult search may illuminate the limits of peripheral vision, not limits on attention

Change blindness refers to the difficulty detecting a change to an image or scene. In the lab, a common experimental paradigm alternates between two versions of an image while introducing a brief blank frame between the pair in order to disrupt motion cues (Rensink et al., 1997). The phenomenon is related to difficulty spotting the differences between side-by-side images in childhood puzzles (Scott-Brown, Baker, & Orbach, 2000).

Many researchers have interpreted change blindness as probing the limits of perception or memory without attention (e.g.. Hollingworth & Henderson, 2002; O’Regan, 1992; O’Regan, Rensink, & Clark, 1999; Rensink et al., 1997; Scholl, 2000). Supposedly, the observer manipulates a spotlight of attention, and perception is richer within that spotlight than outside of it. The difficulty detecting a change appears to imply that little perception occurs without attention.

However, others have suggested that change blindness might be due in part to peripheral vision; visual processing that occurs in the part of the visual field outside the foveola. Peripheral vision is known to be poor relative to foveal vision; visual acuity, contrast sensitivity, color vision, and motion perception all vary with eccentricity (i.e., with distance from the center of gaze; see Rosenholtz, 2016, for a review). A more consequential difference concerns peripheral vision’s degradation in the presence of clutter, known as crowding. The phenomenon of visual crowding illustrates that loss of information in the periphery is not merely due to reduced acuity. In classic demonstrations, observers easily identify an isolated target letter in the periphery, but have difficulty recognizing the target when flanked closely by other stimuli, such as other letters. An observer might see the crowded letters in the wrong order, they might not see the target at all, or they might see a confusing jumble of shapes made up of parts from multiple letters (Lettvin, 1976). Crowding occurs with a broad range of stimuli (see Pelli & Tillman, 2008, for a review). It need not involve an individuated “target” and “flankers” per se, but rather can occur in peripheral perception of complex objects and scenes (Martelli, Majaj, & Pelli, 2005). The degree of difficulty an observer has in making sense of peripheral stimuli varies considerably with the stimulus and task (Andriessen & Bouma, 1976; Kooi, Toet, Tripathy, & Levi, 1994; Livne & Sagi, 2007; Manassi, Sayim, & Herzog, 2012; Sayim, Westheimer, & Herzog, 2010), making it difficult to gain intuitions about the likely impact of crowding in a given situation.

At any moment during a change-detection experiment, the changed region likely lies in the peripheral visual field. This raises the question of whether observers have difficulty detecting changes primarily because of poor peripheral vision. Hard changes might be difficult to perceive in the periphery, whereas one might detect easy changes even without an eye movement. If so, change blindness might not probe the mechanisms of attention so much as it probes the limits of peripheral vision. Various researchers have found evidence for this hypothesis. Henderson and Hollingworth (1999) showed that observers are more likely to detect the change once they have fixated on or near it. O’Regan, Deubel, and Clark (2000) similarly found that probability of detection depends upon the distance between observer fixations and the change. Parker (1978) and Zelinsky (2001) also found evidence that observers can notice at least some changes in the periphery, and that salient changes can even be detected without fixation, in line with the idea that peripheral vision might facilitate detection of easy changes.

**Fig. 2:**
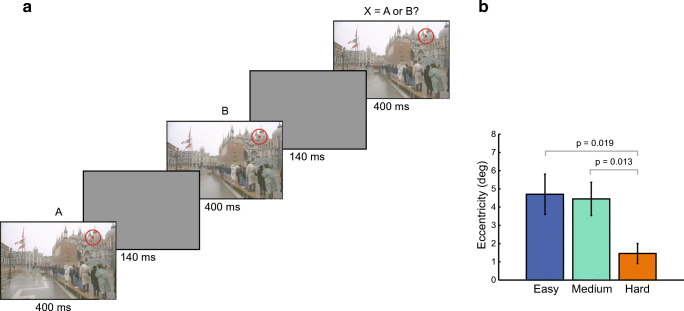
Peripheral vision is a factor in change blindness. A. Observers discriminated known changes in an A-B-X paradigm that requires them to identify whether the final image matches the first or the second image in the sequence. Fixation was enforced at various distances to the change. The red circle shows one such fixation. The difference for this sequence was in the pattern on the ground. B. The threshold eccentricity (distance to the change) for easy-, medium-, and hard-to-detect changes. Harder changes require closer fixation in order to be discriminated.

We have found additional evidence that peripheral vision is a factor in change blindness. We first measured change-detection performance for a number of image pairs, using a standard flicker paradigm (Rensink et al., 1997). These pairs included a number of examples from previous studies of change blindness from Rensink and colleagues. Based on this data, we categorized these change blindness stimuli as easy, medium, and hard, based on the time needed to detect the changes. We then showed observers the changes, and directly assessed the difficulty detecting each change at various eccentricities. As the observer knew each change, they presumably covertly attended to the changed portion of the image when performing this task (see Fig. 2a). We found that for the hard changes, observers needed to fixate significantly closer to the change in order to perceive it (see Fig. 2b; Smith, Sharan, Park, Loschky, & Rosenholtz, 2019). Changes that are harder to detect in a flicker paradigm are harder to see in the periphery, even when observers know the change and its location, and presumably attend to the change. These results suggest a more tenuous connection between change blindness and attentional limits; change blindness may have probed the limits of peripheral vision. Furthermore, they suggest that peripheral vision must guide search for changes. Some changes are easy to detect because they are easily discriminable even at 10 degrees from fixation (Smith, Sharan, Park, Loschky, & Rosenholtz, 2019). Other changes are hard to detect because one must fixate close to the change to reliably discriminate it. For the hard changes, one must move one’s eyes until they get close enough, which will often be a slow process. If observers were not using peripheral vision at all—they just scanned without peripheral guidance until fixating the change—then we would not have found an association between threshold eccentricity for change discrimination and change detection difficulty. In this sense, change detection must occur across the visual field; in parallel, though the observer may not be aware of looking for changes in the periphery. This is in agreement with the suggestion from (Wilken & Ma, 2004) that change detection occurs in parallel. As we will see, this paradigm shift in thinking about change blindness has significant implications for the awareness puzzle.

We have similarly reexamined visual search. In the classic view, search experiments probe limits of attention (Treisman & Gelade, 1980). By comparing conditions that lead to difficult versus easy visual search, we supposedly determine at what stage selection occurs, and what processing is preattentive. Experiments have generally shown that search is difficult whenever distinguishing the search target from other distractor items requires more than a simple basic feature such as color or motion. On the other hand, easy search for simple 3-D shapes and direction of shadows has suggested that the notion of basic features may be more complicated (Enns & Rensink, 1990a, 1990b; Rensink & Cavanagh, 2004). This caveat aside, search results have been taken to suggest that only basic features—often referred to as *feature maps—*can be computed preattentively, and that selection occurs early in visual processing (Treisman & Gelade, 1980). Basic feature maps, without correct binding, and without access to higher level processing (Treisman & Gelade, 1980), can neither obviously support the ease with which vision understands complex scenes nor the power of real-world vision. It is for just this reason that some researchers supplemented their theories with additional pathways or modes for dealing with scene processing (Nakayama, 1990; Treisman, 2006; Wolfe et al., 2011).

However, considerable research has suggested that peripheral vision plays a significant role in search difficulty. If so, at minimum, most search experiments have a peripheral vision confound. Carrasco and colleagues found eccentricity effects in search, leading them to question the role of attention: Both feature and conjunction search deteriorate with increasing target eccentricity, and set size effects become more pronounced (Carrasco & Frieder, 1997; Carrasco, Evert, Chang, & Katz, 1995; Carrasco, McLean, Katz, & Frieder, 1998; Carrasco & Yeshurun, 1998). These effects are eliminated or reduced when the stimuli are M scaled to reduce peripheral factors (Carrasco et al., 1995; Carrasco et al., 1998). Peripheral discriminability of Gabors in noise predicts search for Gabor targets (Geisler, Perry, & Najemnik, 2006). There have also been hints that search difficulty stems from crowding in peripheral vision (Erkelens & Hooge, 1996; Gheri, Morgan, & Solomon, 2007).

**Fig. 3:**
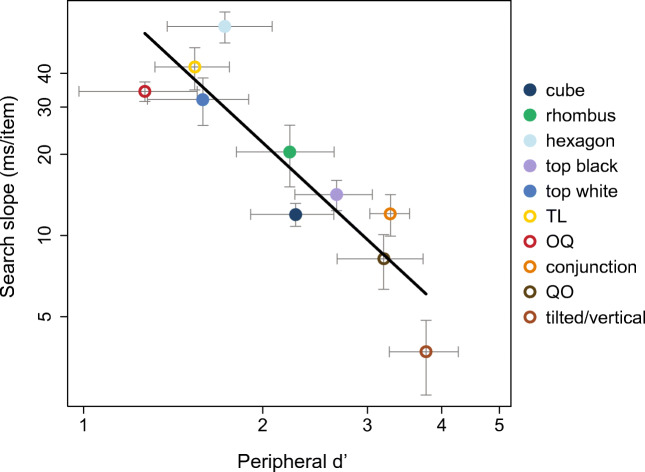
Peripheral discriminability of a crowded target-present vs. target-absent patch (x-axis) predicts search difficulty (y-axis, measured as the slope of the function relating search reaction time to the number of display items). Target-present patches consist of a target flanked by a number of distractors, whereas target-absent patches consist of a distractor flanked by additional distractors. Each symbol represents a different search condition, including both five conditions central to feature integration theory and five problematic conditions showing unexpectedly easy search for a shaded cube among differently shaded cubes. Figure reproduced with permission from (Zhang, Huang, Yigit-Elliot, & Rosenholtz, 2015).

As in the case of change blindness, we have extended this work on search and crowding by having observers attend to the periphery and perform peripheral discrimination of a crowded target-present from a target-absent patch. We have shown that this peripheral discriminability predicts search performance (see Fig. 3). Importantly, many of the phenomena that originally motivated classic selective attention theory are already present in peripheral vision under conditions of crowding. Even when an observer attends to the periphery, they have trouble distinguishing a crowded “T” from a crowded “L.” They perceive illusory conjunctions, reporting the presence of a white vertical when the display had only white horizontals and black verticals. On the other hand, easy search tasks correspond to easy peripheral identification. Peripheral vision preserves the necessary information to identify unique basic features. Peripheral discriminability also explains results on the some of the cube search conditions of (Enns & Rensink, 1990a), which were problematic for classic FIT. The strong relationship between search performance and peripheral discriminability, across a wide range of conditions, suggests that relative search difficulty primarily pinpoints loss of information in peripheral vision, rather than attentional limits or the limits of preattentive processing (Chang & Rosenholtz, 2016; Rosenholtz, Huang, Raj, Balas, & Ilie, 2012; Zhang, Huang, Yigit-Elliot, & Rosenholtz, 2015).

Neither search nor change blindness clearly support classic selective attention theory. Rather, the differences between easier and more difficult conditions may arise from limits in peripheral vision. (This is not to say that search and change blindness encounter no other limits, a point this paper returns to later.) One might ask why this distinction matters, since either explanation implies a loss of information, whether from not attending to a region or from not fixating it. At first glance, either theory would appear to suggest impoverished vision. However, a peripheral vision explanation implies that perception is richer than previously thought. In the classic selective attention explanation, unselected stimuli receive virtually no further processing beyond the bottleneck of attention. Attention, after all, supposedly gates access to higher level processing (Treisman & Gelade, 1980). This means that many, if not most, tasks are impossible without attention, because they require more than the basic feature maps. It was precisely the impoverished vision resulting from classic selective attention theory that led some researchers to add extra pathways and modes to make vision work (Nakayama, 1990; Rensink, 2000; Treisman, 2006; Wolfe et al., 2011). On the other hand, according to the peripheral vision account, difficult change detection and search tasks have relied on information that happens to be lost in peripheral vision; these tasks may be especially difficult, and not imply impoverished vision overall. (This conclusion is in agreement with Rensink’s (2000) theory, which suggested that change detection might be especially difficult—in his case, due to the volatile nature of proto-objects—and might not, as a result, point to impoverished vision more generally.) Peripheral vision preserves much information, and critically, processing continues. Just what information is preserved, and what tasks that information supports, can best be answered with a model of peripheral vision (see the following section).

### A summary statistic encoding in peripheral vision determines difficulty for a range of visual tasks

My lab has argued since 2007 that peripheral vision encodes its inputs in terms of a rich set of image statistics. The term *image statistics* refers to statistics either computed over the pixels of the image or over the outputs of image processing operations, such as filters and nonlinear operators applied to the image. These statistics are *summary statistics*, meaning they pool information over sizeable local regions. These regions grow with the distance to the point of fixation (i.e., the eccentricity). For our candidate model (Balas, Nakano, & Rosenholtz, 2009), we chose as our set of image statistics those from a state-of-the-art model of texture appearance from Portilla and Simoncelli (2000): The marginal distribution of luminance; luminance auto correlation; correlations of the magnitude of responses of oriented V1-like wavelets across differences in orientation, neighboring positions, and scale; and phase correlation across scale. This seemingly complicated set of parameters is actually fairly intuitive. First, the model computes a V1-like representation consisting of a number of feature maps: response to horizontal, vertical, and oblique feature detectors at a number of different scales. Then, in a second stage, the model pointwise multiplies pairs of these feature maps, and then averages over each local pooling region. Essentially, instead of determining at each location in the visual field whether, say, there is a corner composed of a horizontal and a vertical orientation, the model summarizes a bigger area by correlating horizontal and vertical over the entire pooling region; it asks whether horizontal stuff tends to be near vertical stuff. We call this model the texture tiling model.

This encoding leads to significant loss of information, and we have accumulated extensive evidence that this loss of information can predict difficulty recognizing peripheral objects in cluttered displays or scenes (Balas, Nakano, & Rosenholtz, 2009; Chang & Rosenholtz, 2016; Freeman & Simoncelli, 2011; Keshvari & Rosenholtz, 2016; Rosenholtz, Huang, Raj, Balas, & Ilie, 2012; Zhang, Huang, Yigit-Elliot, & Rosenholtz, 2015). The loss of information also predicts difficult search conditions, while preserving the information necessary to predict easy *pop-out* search (Chang & Rosenholtz, 2016; Rosenholtz, Huang, Raj, et al., 2012; Zhang et al., 2015).

**Fig. 4:**
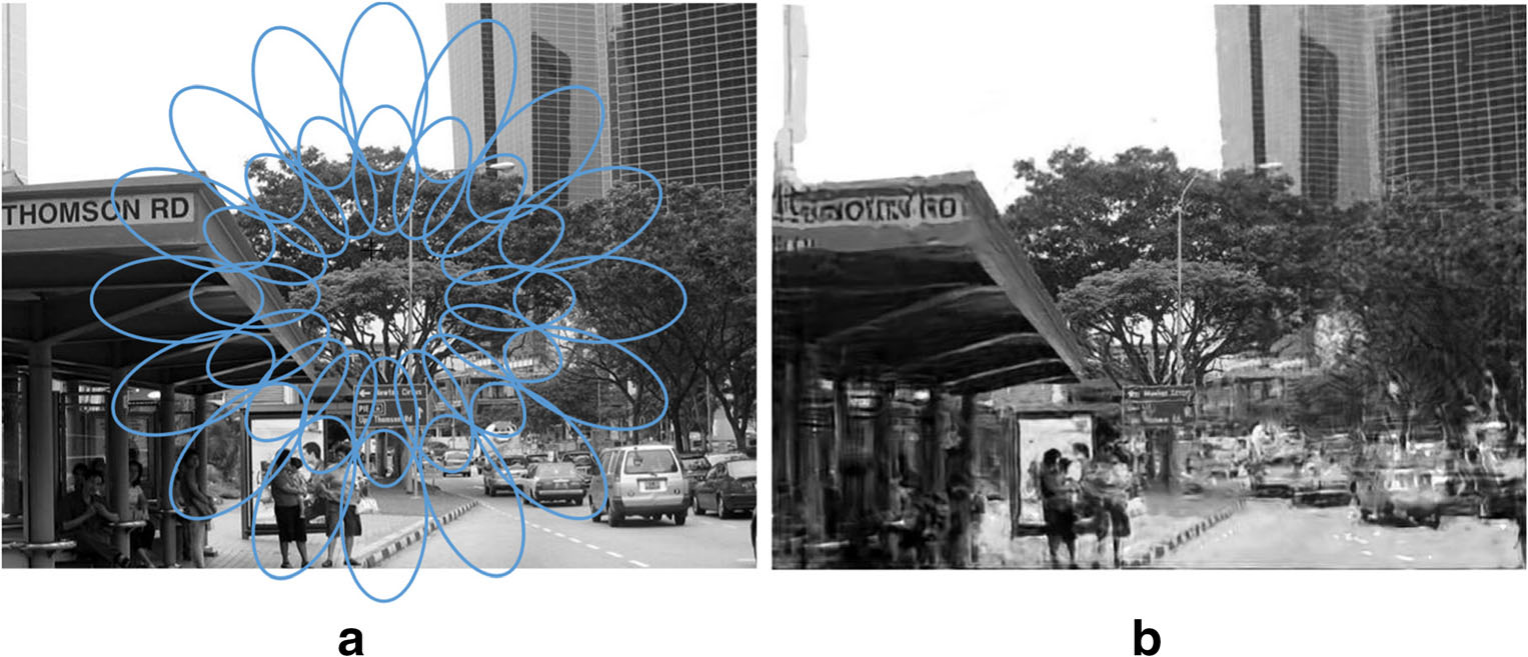
Information encoded by a rich set of image statistics. A. Original image, theoretical pooling regions superimposed. They grow linearly with eccentricity. B. Image synthesized to have approximately the same local image statistics as the original. This encoding captures a great deal of information, although some of the details are unclear.

In spite of the loss of information that leads to crowding, this encoding preserves a great deal of information. To get a sense of what information is encoded by a rich set of image statistics such as those proposed, one can synthesize images that contain the same statistics but are otherwise random (Ehinger & Rosenholtz, 2016; Freeman & Simoncelli, 2011; Rosenholtz, 2011; Rosenholtz, Huang, & Ehinger, 2012). We have called these syntheses *mongrels*. The measured summary statistics do not completely constrain the input, so each combination of fixation and stimulus theoretically corresponds to a number of mongrels, each with the same local summary statistics. One should not think of these images as “what the world looks like to peripheral vision.” Rather, viewing the mongrels (e.g., see Fig. 4), provides intuitions about the information lost and maintained by the peripheral encoding. If some information is clear in the mongrels, then according to the model, that information should reliably be available in peripheral vision. The encoding appears to preserve considerable information about the fact that the underlying image in Fig. 4a is a street scene, with people waiting at a bus stop. Detailed information survives about the appearance of the buildings and trees, and about the general layout of the scene. By asking observers to perform scene tasks with these mongrel images, we have demonstrated that the information encoded quantitatively predicts human performance getting the gist of the scene at a glance. This includes identifying the scene category, upcoming turns, presence of a target object like an animal or a stop sign, and what city appears in the photograph (Ehinger & Rosenholtz, 2016; Rosenholtz, Huang, & Ehinger, 2012).

It is not surprising that the encoding preserves so much useful information, as this scheme involves measuring a large number of image statistics—as many as 1,000 per pooling region. Whereas a handful of summary statistics would not support the richness of vision—consider how little one knows about a scene from only, say, the mean size and color of its items—the proposed encoding is no mere handful. Vision science has done little to characterize our rich subjective impression of the world, but it seems plausible that this encoding scheme preserves enough information to support that percept.

**Fig. 5:**
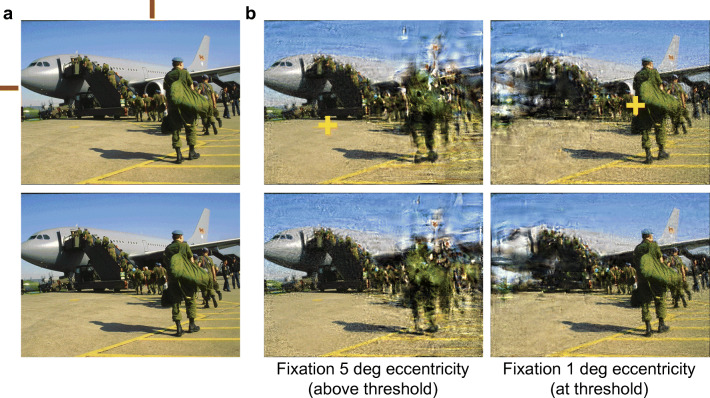
Summary image statistics lose information about the details, which could make change detection difficult. A. Image pair. Red bars indicate changed region: the airplane engine present in the upper image but absent in the lower. B. Mongrel visualizes the information available in a summary statistic encoding for a fixation 5 degrees (left) and 1 degree (right) from the change, as indicated by the yellow cross. Note that the change is clear in the latter pair, but not the former.

By examining Fig. 4, however, it is clear that the encoding does not preserve certain details. One cannot read the Thomson Rd. sign, nor easily discriminate the number and types of vehicles. This ambiguity of the details could underlie poor performance in change-detection experiments (Cohen et al., 2016; Freeman & Simoncelli, 2011; Smith et al., 2019). Figure 5 shows a demo of this same synthesis technique applied to a change-detection pair. When fixating 5 degrees away from the change, the model predicts difficulty detecting that change. However when fixating 1 degree away, the change becomes clear, in agreement with our data on discrimination of this change in the periphery (Smith et al., 2019).

A summary statistic encoding in peripheral vision, then, seems promising in terms of providing a coherent explanation of a number of diverse phenomena that have previously defied easy explanation. The same encoding predicts relative difficulty of different visual search conditions, as well as scene perception performance. Peripheral vision is clearly a factor in change blindness. While further work (in progress) is necessary to test to what degree the model can *quantitatively* predict change-detection difficulty, demonstrations of the information available appear to be in line with difficult change detection (see Fig. 5), and extensive work, cited above, validates the ability of this encoding to predict peripheral discriminability for a considerable range of conditions.

### Comparing the proposed encoding scheme to other theories

**Fig. 6:**
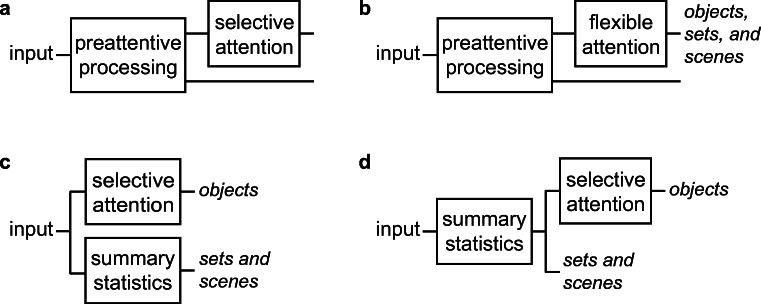
Architecture comparison. These diagrams illustrate only the main blocks associated with hypothesized bottlenecks. A. FIT (Treisman & Gelade, 1980). B. Theories with flexible allocation of attentional resources or modes of attention, e.g. (Nakayama, 1990; Van Essen, Olshausen, Anderson, & Gallant, 1991; Treisman, 2006; Franconeri, Alvarez, & Cavanagh, 2013). C. Theories with separate pathways for scene and set perception, e.g. (Rensink, 2000; Wolfe, Vo, Evans, & Greene, 2011; Cohen, Dennett, & Kanwisher, 2016). D. At minimum, research on peripheral vision indicates that the summary statistic encoding underlies both the selective attention pathway and perception of scenes and sets.

At this point, it is worth revisiting some of the previous theories discussed in the first section. Several proposed an architecture with multiple pathways operating in parallel (see Fig. 6c)—one for selective attention, and one or more in which summary statistics support scene and set perception (Alvarez & Oliva, 2008; Cohen, Dennett, & Kanwisher, 2016; Haberman & Whitney, 2011; Oliva & Torralba, 2006; Rensink, 2000; Wolfe et al., 2011). Other theories (e.g., Treisman, 2006) posit flexible attentional modes, with diffuse attention leading to computation of summary statistics to support scene and set perception (see Fig. 6b). These proposals should sound like (and in the case of Cohen et al., 2016, were at least partially inspired by) our model of peripheral vision. However, our work on peripheral vision suggests several important modifications to these theories.

#### Implications of the peripheral encoding: Rethinking the architecture

The multiple pathway architecture implies that the scene/set pathway(s) have access only to summary statistics, while selective attention provides additional information. However, our model predicts the peripheral information available *when covertly attending* (Balas et al., 2009; Chang & Rosenholtz, 2016; Freeman & Simoncelli, 2011; Keshvari & Rosenholtz, 2016; Rosenholtz, Huang, Raj, et al., 2012; Zhang et al., 2015). Covert attention does not provide information beyond that available in the summary statistics. At minimum, the summary statistic encoding stage must precede both selective attention and scene/set pathways (see Fig. 6d). If one prefers a single pathway architecture with different attentional modes (see Fig. 6b), then the summary statistic encoding must underlie all modes.

Do scene or set processing belong in a special pathway? The question is not whether at some point the visual system carries out different computations when processing a scene versus an individual object; in some sense, this must be true. Visual attention researchers split scenes/sets into separate pathways because those processes seemed *subject to different limits*. Our results suggest, however, that scenes and sets do not deserve special status. Researchers added an additional, statistical pathway to account for good performance on scene and set perception tasks, and in fact, a summary statistic encoding does seem promising at predicting performance at those tasks. However, that same encoding can also predict easy versus difficult search, and likely change blindness; phenomena that allegedly arose from limitations of the selective pathway (Rensink, 2000; Wolfe et al., 2011), or from a focused attention mode (Treisman, 2006). It would seem that search, change blindness, and scene perception might be subject to the same limitations, calling into question the need for multiple pathways with different capacities (as in Fig. 6c–d). Our new understanding of peripheral vision demands rethinking capacity limits.

#### Summary image statistics versus ensemble statistics

The proposed encoding measures a large number of summary *image* statistics, across the field of view, regardless of the contents of the visual stimulus (see also Freeman & Simoncelli, 2011; the texture descriptors of Wolfe et al., 2001; the receptive field-based computation of summary statistics described in Utochkin, 2015; and the large number of image statistics hypothesized to underlie the gist of a scene in Oliva and Torralba, 2006). At minimum, a number of previous proposals have lacked clarity on these points. First, summary image statistics are not the same as *ensemble* properties of a set of *items* (Ariely, 2001; Cohen et al., 2016; Haberman & Whitney, 2011; Treisman, 2006). Ensemble properties refer to summary statistics such as the mean size of a set of items. Summary image statistics, on the other hand, refer to summary statistics computed over the outputs of image processing operations such as filters and non-linear operators applied to the image. Second, some researchers have proposed that ensemble properties represent only certain portions of the visual world (Cohen et al., 2016)—for example, only sets of similar items, or only textures, broadly construed (Haberman & Whitney, 2011; Treisman, 2006; Whitney & Leib, 2018). Third, some previous proposals have implied that the encoding involves only a small number of summary statistics (e.g., Ariely, 2001; Cohen et al., 2016; Haberman & Whitney, 2011; Treisman, 2006).

**Fig. 7:**
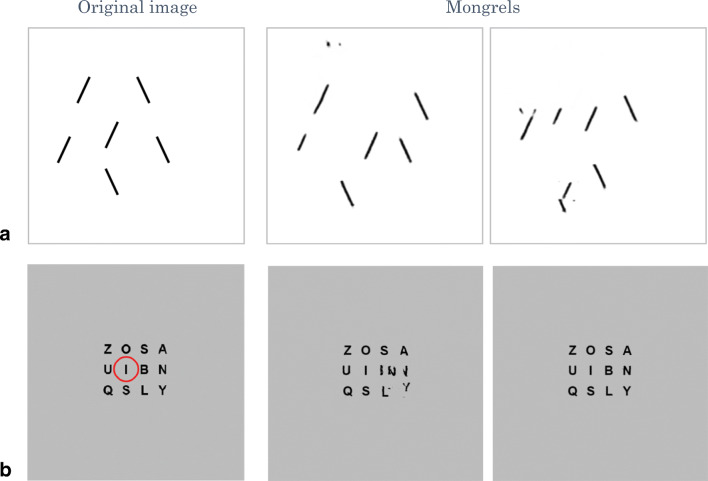
The proposed set of summary image statistics encode considerable information about sets of similar items. A. Original set of oriented lines (left), and two mongrels visualizing the information available (right). Modeled with the fixation 10 degrees to the right of the central target, where each line is 1 degree in length. B. Array of letters (left) like that in Sperling (1960). Mongrels (right) predict that peripheral vision can discern the structure and appearance of the array, and even support identifying the majority of the letters. In the mongrel on the right, reproduction is almost perfect. Fixation on the letter “I”, as indicated by the red circle.

Though summary image statistics and ensemble properties of a set of objects are often confused, there exists an important asymmetry between the two. A large set of image statistics can support not only a variety of scene-perception tasks (Ehinger & Rosenholtz, 2016), but also plausibly form the basis for ensemble perception tasks (see Fig. 7a; although see Balas, 2016, for questions of whether our particular candidate encoding can quantitatively predict judgments of numerosity). In contrast, a handful of ensemble statistics cannot obviously support rich scene perception, and without specifying the statistics, it is not even clear that they can support the rich perception of ensembles. As Huan, Tononi, Koch, and Tsuchiya (2017) point out, referring to an array of letters (see Fig. 7b), observers likely know quite a bit about ensembles:

Is that really all they see, [3–4 items] perhaps augmented by some summary statistics? A moment’s reflection indicates that, if only they were asked, subjects could report much more—one certainly perceives that there are many black marks, that they are arranged in rows and columns, in a rectangular array, . . . against a bright homogeneous background . . . [these percepts are] typically taken for granted rather than included in the catalog of conscious contents. . . . While subjects may not be able to recognize specific identities, . . . they can effortlessly report that what they saw were letter-like figures. p. 3.

A critical point here, however, is that while “some [unspecified] summary statistics” cannot obviously predict this rich percept, a set of many summary statistics can. As the mongrels in Fig. 7a show, the proposed encoding clearly preserves sufficient information to answer questions about the distribution of line orientations, including the mean and variance. In addition, it preserves enough information to tell that the stimulus is composed of black marks on a light background. The sizes and orientations of items are also largely preserved, but location information is lost; the lost information perhaps partially explains the difficulty reporting the features of a *particular item* (e.g., in Fischer & Whitney, 2011; Parkes, Lund, Angelucci, Solomon, & Morgan, 2001). The mongrels of the letter arrays (see Fig. 7b) similarly indicate that the encoding preserves precisely the sort of information enumerated by Huan, Tononi, Koch, and Tsuchiya (2017). In addition, it appears that sufficient information survives to recognize 10–12 of the letters—far greater than the average 4.3 items available for immediate report, but comparable to the 9.1 letters estimated to be available by partial report (Sperling, 1960).

Perhaps some previous theories have described the representation of ensemble statistics instead of image statistics as merely a rhetorical figure of speech. It is probably easier to get intuitions about and to enumerate the mean size and orientation of a set of items than to think about more abstract image statistics. In addition, researchers may have inadvertently implied that their theories required only a few statistics because of the difficulty coming up with a long list of plausible ones. Both points, however—image statistics, and lots of them—are critical to the argument that such an encoding could underlie the richness of perception. It is important to be explicit. When Cohen et al. (2016) refer to a “single summary statistic” (p. 325), this could refer to a single high-dimensional vector—which is, after all, what underlies their demos from Freeman and Simoncelli (2011) and from Oliva and Torralba (2006)—but if so, they risk confusing their readers.

**Fig. 8:**
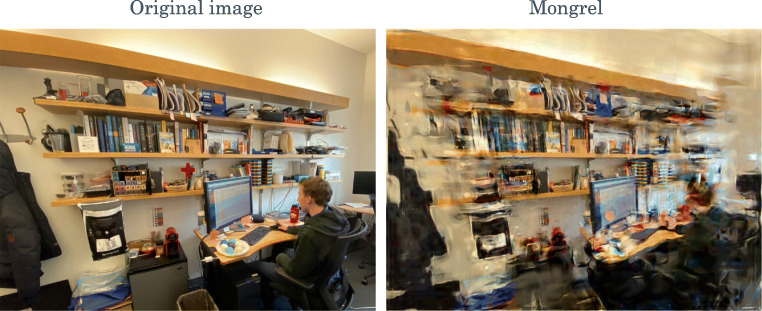
Looking for one’s mug on the desk, the same peripheral encoding that predicts difficult search and change blindness provides ample information to locate the desk, notice salient objects, and guide eye movements to gather additional information. Fixation as indicated by the red cross at image center.

Returning to the question posed in the second section of this paper: Does it matter, when asking whether vision is impoverished, if tasks like search and change blindness are difficult because of the limits of attention or the limits of peripheral vision? Clearly it does. The classic selective attention explanation requires an additional mechanism, such as an added pathway or attentional mode, to explain why observers easily get the gist of scenes and sets, whereas the peripheral vision explanation does not. An added gist pathway or attentional mode might have solved Mack and Rock’s (1998) chicken-and-egg problem of how one can successfully direct attention (Oliva & Torralba, 2006; Rensink, 2000; Wolfe et al., 2011). However, with our model of peripheral vision in hand, we can do better, by making concrete predictions of what information is available. Consider looking for one’s mug in the office scene in Fig. 8. Starting with a central fixation, the proposed encoding scheme provides ample information for locating the desk and noticing salient pink sticky notes. It may provide enough information to notice the student sitting at the desk, although perhaps not, since his shirt blends in with the chair. The first glance may not preserve enough information to find the mug (gray, on the desk behind the monitor). One cannot recover the information lost in peripheral vision without an eye movement, but the information that remains is capable of supporting performance of many tasks, from guiding eye movements, through some object recognition tasks, to getting the gist of a scene and navigating the world.

## A proposal for an additional capacity limit: Limited decision complexity

### Other difficult tasks may be inherently difficult

Given the strengths of peripheral vision, it is not surprising that observers can easily get the gist of a scene or set. The limitations of peripheral vision, on the other hand, can explain many of the phenomena previously taken as evidence that perception is poor without attention. This paper began, however, by also enumerating a second set of phenomena that suggest that attention itself is limited, as is visual working memory (see Fig. 1). One cannot explain these phenomena using peripheral vision alone. Peripheral vision could be a *factor—*inattentional blindness, multiple object tracking (MOT), and visual working memory (VWM) tasks often use crowded displays, and typical dual-task experiments assign one task to peripheral vision. However, a number of inattentional blindness studies (Levin & Simons, 1997; Mack & Rock, 1998) have enforced fixation and have found that knowing the task matters. Visual working memory studies (e.g., Adam, Vogel, & Awh, 2017; Tamber-Rosenau, Fintzi, & Marois, 2015) have controlled for peripheral crowding and found similar memory limits. Typical dual-task experiments (e.g., VanRullen et al., 2004) hold fixation and the display constant, and vary the number of tasks; though peripheral discriminability does appear to be a factor in the relative difficulty of a given dual task (Rosenholtz, Huang, & Ehinger, 2012), it cannot explain why many dual tasks are more difficult than their component single tasks. Other tasks may also encounter additional limits; search and change detection, for instance, may be more difficult than predicted from peripheral vision alone, due to the need to perform peripheral discrimination of a number of different items (Rosenholtz, 2017). There must be some other capacity limit(s).

It may be tempting, at least in the case of dual-task performance, inattentional blindness, and MOT, to fall back on selective attention theory to explain these results. However, quite a bit of the evidence for the classic selective attention theory had a peripheral vision confound, and peripheral vision offers a more parsimonious account, since it predicts easy scene perception as well as difficult search and change blindness. Given that search difficulty may have pinpointed mechanisms of peripheral vision rather than of early selective attention and preattentive processing (Treisman & Gelade, 1980), we must reconsider the need for preattentive maps of basic features and for a serial selective mechanism to bind them (Chang & Rosenholtz, 2016; Rosenholtz, Huang, Raj, et al., 2012; Zhang et al., 2015). Even given a need for a serial mechanism of some sort, at minimum it would seem a useful exercise to start from scratch in examining the remaining capacity limit(s). For further arguments for why we need to look for a different sort of capacity limit, and for different mechanisms for dealing with that limit, see Rosenholtz (2017).

Of course, there could be no unifying explanation for MOT, VWM, dual-task performance, and inattentional blindness. MOT might face limits on, say, the number of attentional spotlights, VWM on the number of memory slots, dual-task performance and inattentional blindness on the simultaneous tasks one can perform. However, perhaps we can arrive at a unifying explanation by noting commonalities among these tasks that suffer additional limits.

Consider a typical VWM task. An observer views an array of *k* items, such as colored disks (see Fig. 1, lower right). After a delay, the experimenter then presents another array that either duplicates the original, or differs in the color of one of the *k* disks. (The VWM paradigm sometimes instead asks the observer to specify the features of a particular postcued item. For the sake of argument, I assume that changing the task in that way does not fundamentally change its difficulty nor the mechanisms involved.) This task would be easy if the brain were like a computer, storing either the pixels from the previous stimulus, or each item in its own memory slot; the observer would simply compare the later display to the stored representation to detect that one of the items had changed. However, performance suffers when displays contain more than a few items, leading to the traditional interpretation that observers only have access to about four slots, suggesting a very limited VWM capacity (Luck & Vogel, 1997).

This logic, however, makes strong assumptions about the mechanisms underlying VWM. More generally, one might think of the VWM task as setting up a classifier to distinguish between the remembered stimulus and all other similar arrays in which one item differs. If we more generically think of the representation of the observed array of items as some noisy high-dimensional feature vector, one could imagine that the task might be difficult to perform using, say, a simple linear classifier. A very similar story applies to tasks such as reporting a postcued member of an ensemble—essentially a VWM task, and likely hard at least in part for the same reason.

Similarly, MOT tasks (see Fig. 1, upper left) might be easy if the brain were like a computer. If the brain stored one pointer per display item, observers would just need to update the pointer for each target with its location in each subsequent frame. However, observers generally have difficulty tracking more than four targets, leading to the traditional interpretation that the visual system only has about four attentional spotlights to deploy, suggesting that attention has limited capacity (Pylyshyn & Storm, 1988).

However, as with VWM, this account makes strong assumptions about the mechanisms involved. More generally, if the observer must track *k* of *n* items, then on each frame they must distinguish the actual *k* targets from *n*–choose–*k* other possible combinations of *k* items. Again, one might imagine that this discrimination might require a complex classifier. In the case of tracking 4 of 9 items, for instance, the observer must distinguish the actual four targets from 125 other possibilities! In the abstract, this sounds inherently difficult, though, of course, motion cues make the task more tractable.

**Fig. 9:**
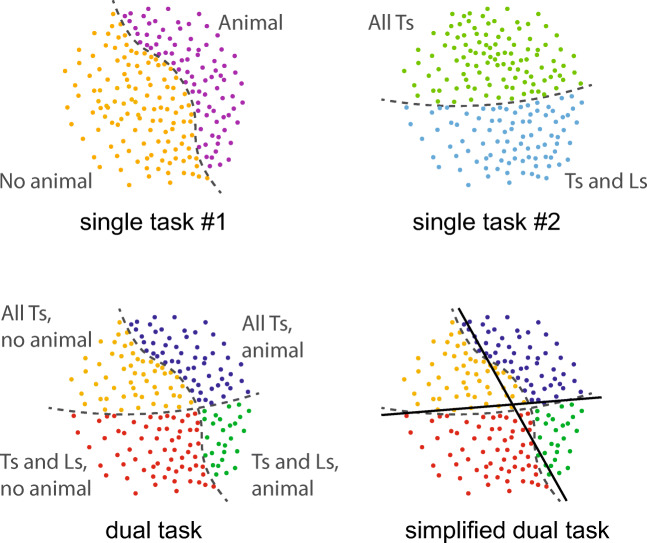
Dual tasks are inherently more complex than their component single tasks. Here, two 2AFC tasks (top) become a 4AFC dual task (bottom left). If there exists a limit on task complexity, the observer will have to simplify this task (bottom right, solid lines), making errors.

Consider also typical dual-task experiments (e.g., VanRullen et al., 2004; see Fig. 1, upper right). The observer is asked either to complete a single peripheral task or to perform that task as well as a central task. For instance, the observer might specify whether a peripheral cube is upright or inverted, while also indicating whether a central array contains all the same letter (all “L”s or all “T”s) or different letters (both “L”s and “T”s). Both the central and peripheral task involve distinguishing between two alternatives. The dual task involves distinguishing between four possibilities (see Fig. 9). This renders the classifier needed to perform dual tasks inherently more complex than that needed for the component single tasks. The boundary needed to separate the classes is inherently more complex.

The previous discussion suggests a commonality that—viewed in terms of classification of a noisy feature vector representing the stimulus—MOT, VWM, and dual tasks all appear complex. Nonetheless, observers might be able to perform the tasks, if the visual system could build classifiers of arbitrary complexity. Instead, observers appear limited in the number of items they can encode in VWM, the number of items they can track, and the tasks they can simultaneously perform. This suggests a *limit on decision complexity* (Rosenholtz, 2017), affecting all of these tasks. Limits on decision complexity might originate at a late, decision-level processing stage.

The exact nature of the limit remains unclear. It appears to be a limit on *complexity*, rather than on task *difficulty*. Dual-task experiments have varied the display time so as to make all component tasks equally difficult (e.g., VanRullen et al., 2004); as a result, task difficulty, per se, cannot be the deciding factor for which dual tasks are easy or hard (Rosenholtz, 2017). A limit on task complexity could take different forms (Rosenholtz, 2017). Our cognitive processes might be limited in the number of dimensions (or neurons) one could use to make a decision, in the number of linear hyperplanes out of which one could form a decision boundary, or in the curviness of that boundary. Such a complexity limit might exist for the usual reasons given for capacity limits (e.g., limits on the size of the brain; Tsotsos, 1990). In addition, in learning to perform a classification task, limiting the complexity of the decision boundary might be a way to avoid overfitting.

In fact, some difficult dual-tasks do seem particularly complex, given what we know about peripheral encoding. For example, it is difficult in a dual-task paradigm to judge whether a cube is upright or inverted (VanRullen, Reddy, & Koch, 2004). According to our peripheral vision model, this judgment cannot be made on the basis of a single pooling region, as that representation cannot distinguish between the two orientations; multiple pooling regions are required (Zhang et al., 2015). For the sake of argument, one can loosely think of this as though one pooling region detects the top of the cube, another the bottom, and the two regions together can determine the orientation. This raises the obvious question of why two pooling regions are necessary; can one not just detect the top of the cube with a single pooling region at the location of the top of an upright cube? But that would not suffice if the observer had uncertainty as to the location of the cube. In fact, in the experiment, the cube location varied trial by trial. (Attempting to guess experimental details from the results plus a model provides a useful test of the model.) Either the visual system must make a complicated decision, attempting to detect the top of the cube throughout the visual field and comparing its location to the detected cube bottom, or it must hope that the presentation time is long enough to allow wiring up a cube-orientation classifier on the fly, once its location is surmised.

Not all dual-tasks are so complex. Although they are inherently more complex than their component single tasks, some dual tasks might nonetheless be sufficiently simple that they would be largely unaffected by the complexity limit. This could explain easy (previously dubbed *preattentive*; e.g., VanRullen et al., 2004) dual tasks.

### Limited decision complexity: Implications for a rich subjective impression and real-world vision

Let us consider a couple of related examples, both to get used to thinking about decision complexity and to tie this proposal back to the awareness puzzle and to the success of real-world vision.

#### Is getting a rich subjective impression less complex than remembering four items?

**Fig. 10:**
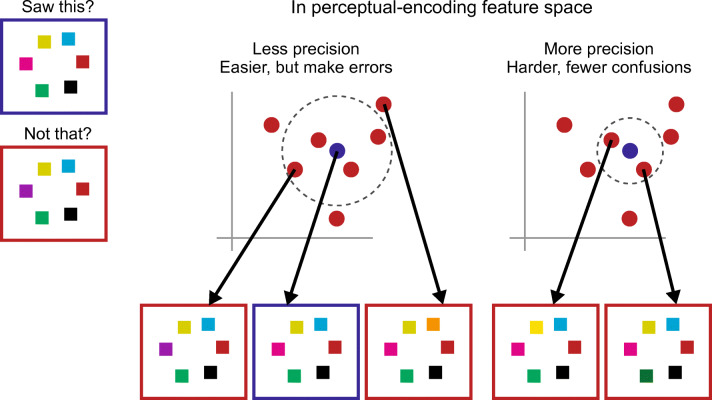
At a basic level, we can think of visual working memory tasks as distinguishing between the observed stimulus and all similar stimuli that differ in one of the items (upper left). If we think of each stimulus image as represented by a high-dimensional vector in some perceptual encoding space (shown here with only two dimensions for simplicity), then we can think of this discrimination as a classification. Dashed lines indicate two possible classification boundaries. The boundary on the right is more precise, distinguishing the observed array (blue) from most other arrays, except those with small color differences. Capacity limits may prohibit such a precise classification, perhaps because they limit complexity, e.g. curvature of the decision boundary. Instead, the brain may be forced to use a less precise decision boundary, such as that shown on the left. This may require less effort, but leads to more significant confusions between the seen and unseen arrays.

The reader could, at this point, have an important question: I have argued that VWM is limited because it is an inherently complex task; how complex, then, is scene perception? In both cases one might think of the implicit task as distinguishing between seen and not seen—essentially as localization in some perceptual encoding space (see Fig. 10). In the proposed theory, what the observer *knows* about the stimulus as a result of performing this localization task—what they *perceive—*is determined by the classification into seen and not seen. If the classification boundary confuses two images then *from this classification task alone* (a point we will consider shortly), the observer cannot perceive the differences between them. Lower precision at this task might require less effort, but at the cost of confusing more unseen stimuli with the one actually seen; with lower precision, the observer knows less. With more effort, the observer might be able to utilize a more complex—higher curvature—classification boundary between seen and unseen stimuli, making fewer errors. However, if there exists a limit on decision complexity, that means that precision and knowledge about the stimulus are limited.

When we speak of a *limit*, this implies the existence of a single cap that all visual tasks must obey. I have been assuming that VWM tasks encounter this limit, making it appear that we can remember only about four items at a time. If our scene perception encounters the same limit, how rich should we expect that percept to be? The answer depends fundamentally on the underlying perceptual encoding, which remains essentially unknown. However, we can get a hint of the answer from the following mini experiment.

Let us take our candidate perceptual encoding from a convolutional neural network (CNN), known as VGG-16, which was trained to perform invariant object recognition in real-world scenes (Simonyan & Zisserman, 2014).^2^ CNNs have recently become very popular, as for the first time they allow computer vision to approach human performance on certain proscribed visual tasks. Researchers have also shown certain similarities between the representations learned by CNNs and those found in monkey physiology (Yamins et al., 2014). On the other hand, issues clearly remain, as CNNs behave differently from humans in a number of ways (e.g., Dodge & Karam, 2016; Geirhos et al., 2019; Geirhos et al., 2018)

We took a set of arrays of eight colored squares against a gray background and fed them into the network to generate a feature vector for each image. For the feature vector, we used the last representational layer (the *last fully connected layer*) of the network; it is common in computer vision to use this layer as the input to classifiers.

**Fig. 11:**
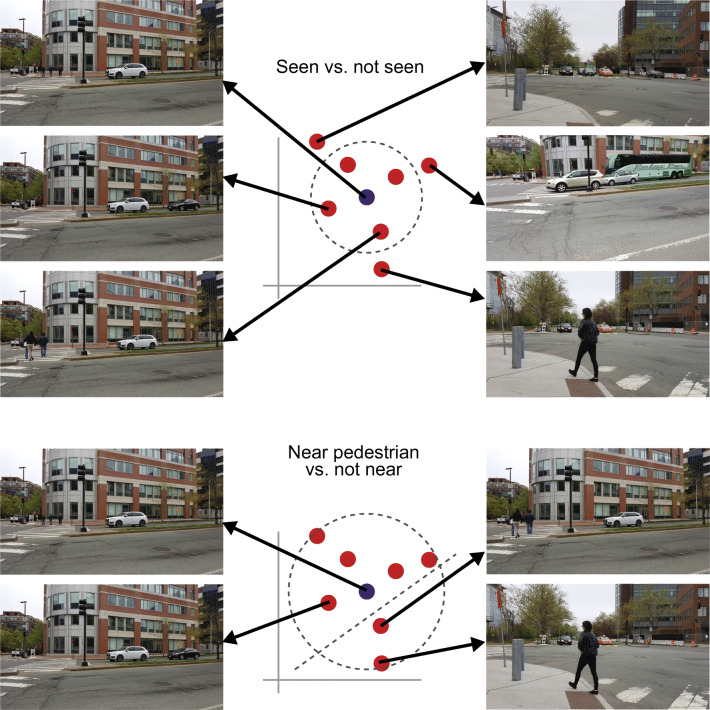
(top) The three confusable images on the left have similar mean discriminability as arrays of 8 colored squares, given the perceptual encoding space of the VGG-16 neural network. The three images on the right are less confusable with these images, according to discriminability in that feature space. (bottom) Switching to a different task can lead to new understanding of the scene. At the next moment, the visual system might attempt to discriminate scenes with nearby pedestrians (right) from those for which the pedestrians were absent or farther away (left).

These images are confusable in a standard VWM task; we can measure the distance between their VGG-16 feature vectors to give us an estimate of the available precision for localizing any image in perceptual encoding space. Given that same uncertainty, how well could we instead pinpoint a natural scene? We took a set of similar street scenes, computed their VGG-16 feature vectors, and then asked what scenes would be difficult to discriminate, given the same precision inferred from the VWM stimuli. The top left of Fig. 11 shows a set of three confusable scenes, according to this metric. However, by this metric these scenes are discriminable from those in the top right.

The first thing to note is that a distance metric applied to the last fully connected layer of VGG-16 seems to give us a reasonable measure of perceptual similarity (at least in this example; given the limitations of CNNs, I would be surprised if this demo worked in general). It is difficult to distinguish arrays of randomly colored squares from each other (see Fig. 10), and analogously difficult to distinguishing the confusable scenes in Fig. 11. Those scenes do differ: The camera angle has changed somewhat, and the location and number of vehicles and pedestrians has changed. The less confusable scenes in the top right appear more readily discriminable. So, the mini experiment is a good first attempt. More importantly, note that for the same amount of uncertainty that makes an eight-item VWM task hard, one can pinpoint a scene fairly well. The *gist* resulting from performing this task appears quite rich and goes far beyond merely categorizing the scene. No doubt the visual system developed to make this so. In a plausible perceptual encoding space, the same precision can specify either “an array of about eight items of random color and position,” or mostly determine the scene, plus or minus some small changes. This suggests there is real hope for a unified explanation. The same inference limits that make VWM difficult allow a rich subjective experience of the real world.

#### Changing the task makes real-world vision work

In real-world vision, we often need to know more about the scene; for example, when driving, we must estimate the 3-D location of the pedestrians in order to judge whether we can turn left. Thankfully, our perception is not limited to the results of performing the *gist* task just described. In the next instant, the observer can perform a different task, i.e., pose another question and make a new inference. In this case, the observer might next ask about the location of the pedestrians (e.g., classify the scene into those containing near vs. far pedestrians). The layout information gained from the gist task provides likely pedestrian locations. The pedestrian localization task, because it does not require detailed knowledge of the rest of the scene, could be less complex. As a result, it might discriminate between near and far pedestrians even if, because of complexity limits, the gist task could not. The observer gains additional understanding about the pedestrians at the expense of comprehension of the scene as a whole. Many typical real-world tasks probably have low complexity relative to the limit—again, the brain has likely developed its representation to make this the case. The visual system may use the excess resources to perform a dual-task: Judge the distance to pedestrians while also getting an impression of the scene as a whole. As a result, an observer estimating the 3-D position of the pedestrian may not completely lose the gist, but may just become more imprecise at localizing the scene in the perceptual encoding space. Nonetheless, observers may not generally run at full capacity, using all the available decision complexity, as doing so may require noticeable effort.

Similarly, in the VWM task, the lack of precision when trying to remember the entire array does not imply that the observer cannot discriminate whether a particular square is red or blue. If that is the task—for instance, if one of the squares is precued (“remember this one”)—then the observer can set up a relatively simple classifier to discriminate the color of that square, again likely at the expense of some details about the set as a whole.^3^ Thinking of visual working memory as a task for which one has flexibility in how to draw the decision boundaries distinguishing seen from not seen clearly has parallels with flexible resource theories of VWM (Fougnie, Cormiea, Kanabar, & Alvarez, 2016; Ma, Husain, & Bays, 2014; Palmer, 1990).

Short-term learning, such as learning to forage for mushrooms, might involve a subtle change in task; fine-tuning of the classifier when one learns to distinguish edible from poisonous, without a change in complexity. Overlearned tasks like reading, on the other hand, might lead to development of representations that simplify those tasks.

#### All perception results from a task, and all tasks encounter the same limits

We should emphasize several important points about visual tasks, from the discussion earlier in this section. First, to talk about decision complexity, there must be a decision. Throughout this discussion of decision complexity, I have assumed that all visual perception arises as a result of performing some visual task; the observer poses a query and makes an inference. Both the query and the inference may be unconscious (Helmholtz, 1867); if the latter is, then perception occurs without awareness. The task may not be the nominal task specified by the experimenter, and, in fact, many real-world visual tasks likely consist of a series of simpler tasks. For a unifying explanation, all visual tasks must face the same limit on decision complexity.

This paper proposes a number of ways of thinking about visual tasks. Many visual tasks, such as visual working memory tasks, can be thought of as distinguishing seen from not seen, which we might think of as a classification task, or as localization in some perceptual encoding space. It may be helpful to think of our “rich subjective experience” as resulting from performing such a localization task. Similarly, it can be useful to think of dual tasks as a single, more complex task. Cueing, different strategies, and knowledge may all lead the observer to pose a somewhat different query—that is, to perform a different task.

### Peripheral vision plus limited decision complexity: Making sense of other phenomena

With a model of peripheral encoding plus a hypothesized limit on decision complexity, to what degree can we understand other phenomena? Of course, a full model of vision requires mechanisms specific to particular tasks and stimuli, but the challenge is to explain as much as possible with minimal additional components.

#### Reframing attentional strategies in terms of decision complexity

In the traditional account, attention allows us to deal with the vast amount of information that confronts us, by prioritizing some aspects of that information at the expense of others. Attention might range in breadth from a focal mode that provides object properties, to a diffuse mode that leads to scene and set properties; it might operate in a single spotlight or with a small number of foci; and observers might attend to a particular object, color, or location (Rensink, 2015). These are intuitive notions of how humans prioritize visual information; how, then, do we think about these concepts in terms of decision complexity?

At a given moment, the complexity of our nominal task may exceed our complexity limit. If the ideal classifier required an overly sinuous decision boundary, the visual system would instead have to perform a simpler task. It could, theoretically, make the task simpler (e.g., the boundary straighter) in many different ways, but would make errors as a result (see Fig. 9, bottom right). Simplifying strategies might include setting up a classifier to give preference to identifying only one object, only objects with a certain color, or only the object at a particular location. The visual system could choose to perform a task that preferentially understands a small set of items at the expense of others; intuitively, the ability to do so would depend on the complexity of the per-item task, the number of items, and their layout. The observer could give preference to understanding an item that lay outside of the fovea. In addition to these strategies, with obvious parallels to object-based, feature-based, spatial, multifocal, and covert attention, respectively, the visual system may have available additional strategies not so easily described in words; ways of *cutting corners—*literally (see Fig. 9)—in order to simplify an overly complex decision boundary.

One can also draw connections to the role of task difficulty in theories of attention. Flexible resource theories suggest that, for instance, the number of items that one can track depends on the resources needed to track each one (i.e., on the difficulty of that subtask; Alvarez & Franconeri, 2007). Similarly, cognitive load—overall difficulty of the current tasks—matters for task performance. In the decision complexity framework, when an experimenter makes a task harder, they often make it more complex. (However, this is not always the case, as a task can be harder by being data limited, which should not increase complexity.) Similarly, we can think of cognitive load manipulations as changing the complexity of the task as a whole. In decision complexity theory, difficulty --in particular, complexity-- has a starring role as the limited resource.

#### Surprising failures in cueing tasks

Many lab tasks explicitly ask observers to selectively process a target. For instance, task instructions may ask observers to identify or report a change only to a cued display item, while ignoring distractors (e.g., Lavie, Hirst, de Fockert, & Viding, 2004; Posner, 1980). Selective attention is the *task*, regardless of the underlying mechanisms. Such cueing tasks often demonstrate a failure to respond only to the target. Lavie et al. (2004), for instance, find significant distractor compatibility effects when the task requires observers to respond as to the identity of the target while also remembering a single digit. This failure to select would be surprising if selective attention were really the main mechanism that the visual system uses to deal with limited capacity; why would such an important mechanism be so flawed? However, completely ignoring the distractors may require a complex classifier, which in turn might require considerable effort. Observers can probably identify the target without completely ignoring the distractors. Why should they put in the effort, if the cost of the distractor compatibility effects is only about 140 ms? The observer may not even realize that they are slower on compatible trials.

#### Blindness to continuity errors in motion pictures and to slow changes

A number of change blindness studies have explicitly studied change to attended and presumably fixated objects (I use “attended” here in the lay sense of “paid attention to”). Levin and Simons (1997) showed subjects movies in which several objects changed between scene cuts and examined how well observers could detect these continuity errors both in normal viewing and when explicitly looking for the errors. In one example, a pair of women sat at a table talking, and between cuts, one woman’s scarf disappeared, the other’s arms moved, and the women’s plates changed. Observers likely paid attention to both of the women; how then, did they not notice changes to the women?

In the first condition, observers did not know that they needed to detect continuity errors. If all perception is the result of some inference, then having a “surprise” task like this can interfere with setting up the “right” classifier, and as a result can reduce the likelihood, in this case, of noticing an unexpected change. When attending to the women, observers probably fixated some part of their faces. The scarf was closest to fixation, followed by arms and then plates. Despite the closeness of the scarf, when the observer does not know about the possibility of continuity errors, they may set up a classifier to get a general awareness of the scene. The result of this task may be a great deal of information about the scene, and yet it may not be sufficient to distinguish between scenes with a scarf and without a scarf. Put another way, to detect the changes one needs to set up a task to discriminate between what one has seen and not seen. Since the observer does not know what details are important, their classifier might not catch the right information. The situation may actually be worse because the observers were told to “pay close attention,” perhaps causing them to choose a more specific task at the expense of understanding the scene as a whole. One would expect that the scarf change would be most noticeable once the observer knows the real task of detecting continuity errors, since it is probably most visible when fixating the face. This is in fact what Levin and Simons (1997) found. The plates may be too far away from fixation to easily notice the change. The arm position, while closer to fixation on the face, may suffer a question of definition: while the experimenters may interpret no time as having passed during the frame cut, the observers may have a different (likely unconscious) understanding. Perhaps observers thought that enough time had passed to allow an arm movement.

One would correctly expect that observers would have even more difficulty detecting gradual changes (Simons, Franconeri, & Reimer, 2000). The observer in this paradigm has the usual problem of setting up a seen versus not seen classifier that happens to distinguish a given change. In addition, the difficulty is amplified by the fact that such changes are gradual enough that it likely takes a significant amount of time for the region to change by more than a just-noticeable difference, and that amount of time is likely longer in the periphery. Most observers probably naturally make several saccades during that time. This gives the observers a difficult task of discriminating what they see in one region of the visual field from what they saw some time before in a different region of the visual field, adding complexity to the task.

#### Inattentional blindness

Again, a surprise task, as in inattentional blindness paradigms, can interfere with setting up the right classifier for that task, and as a result can reduce the likelihood of noticing an unexpected stimulus. Mack and Rock’s (1998) standard paradigm had a hard central task of distinguishing between the lengths of two orthogonal lines. We might presume that this task alone was complex, though perhaps below the complexity limit. As in the previous pedestrian example (see “Changing the Task Makes Real-World Vision Work”), the observer likely attempts to do a dual task that also gets a crude gist of the display. As a result, they may notice some unexpected stimuli, but not all. We would expect that a foveal inattentional blindness task would be even worse, which is, in fact, what Mack and Rock found. They gave the observer the same line-length comparison task, only in the periphery. This task is certainly harder, and probably more complex because of the oddities of the peripheral encoding. Even though noticing an unexpected foveal stimulus should be easy if it were the only task, a more complex primary task in the periphery leaves the observer with fewer resources to devote to the gist of the display, making noticing less likely. With a known dual task, the observer can more appropriately distribute these resources between the two tasks and may even put in more effort to use more of the available decision complexity.

#### Illusory conjunctions

As discussed in the second section, many illusory conjunctions occur in peripheral vision simply due to the nature of the peripheral encoding (see Chang & Rosenholtz, 2016, for an explanation). But what about foveal illusory conjunctions? Treisman and Schmidt (1982) show a list of three colored letters at the fovea, flanked by black digits. Observers must first identify the numbers, and then report the position, color, and identity of the letters. They often make illusory conjunction errors, even though the letters lie in the fovea. From the point of view of peripheral vision alone, this is surprising. Treisman and Schmidt interpret their results in terms of a requirement for attention to correctly integrate features, and the task overloading attention. In the decision complexity explanation, the observer also lacks resources, but of a different kind; the task of simultaneously identifying the central and peripheral symbols is too complex to perform all at once. The observer will make errors. We should not make too much of the tendency to report illusory conjunctions. The researchers varied the display time to set the difficulty level. Make the peripheral task too easy and the observer makes no errors. Make it too hard and they merely guess. Somewhere in between, the difficulty seems just right, and the observer will make the most obvious sorts of errors: reporting an item at the wrong position, and reporting illusory conjunctions.

#### Why are there no tasks that do not require attention?

Under classic selective attention theory, tasks that required only preattentive information were presumed not to require attentional resources. However, researchers have identified few tasks that consistently appear not to require attention. By some accounts, noticing an oddball item (e.g., a moving item among stationary) or getting the gist of a scene might not require attention (Li, VanRullen, Koch, & Perona, 2002; Otsuka & Kawaguchi, 2007; Rousselet, Fabre-Thorpe, & Thorpe, 2002; Treisman & Gelade, 1980). However, even these results have been called into question. Detecting a change has long been considered easy if observers have access to a sufficiently salient motion transient. However, Matsukura, Brockmole, Boot, and Henderson (2011) showed that when performing a secondary task, observers miss changes even when the motion transient is present. Similarly, Cohen, Alvarez, and Nakayama (2011) have shown that getting the gist of a scene becomes difficult in a dual-task paradigm, so long as the secondary task is sufficiently hard (see also Joseph, Chun, & Nakayama, 1997; Larson, Freeman, Ringer, & Loschky, 2014; Mack & Clarke, 2012; Rousselet, Thorpe, & Fabre-Thorpe, 2004). It seems that no tasks categorically require no attentional resources.

These results make sense if we think in terms of decision complexity limits, and consider the two subtasks in a dual-task paradigm as a single, more complex task. If one adds a sufficiently complex task to oddball detection or getting the gist of a scene, one can always make the dual task as a whole encounter complexity limits.

## Additional comparison with existing theories

The section titled “Comparing the Proposed Encoding Scheme to Other Theories” compared our proposed peripheral encoding to previous solutions to the awareness puzzle. The next section compares switching tasks in the decision complexity framework to the concept of changing the allocation of attention from focal to diffuse. The section titled “A Predictive, Testable Theory” discusses an important benefit of the proposed two-part theory. The section “Revisiting Illusion and Inaccessibility Theories” revisits these theories of the awareness puzzle in light of the present hypothesis.

### Comparing limited decision complexity to theories with flexible modes of attention

Treisman (2006) suggested that attention is a limited resource with some flexibility in how diffusely it can be allocated. Attending to a scene or a set yields holistic properties without the details, whereas object-based attention yields understanding of the object at the expense of the scene. Other researchers have made related proposals (e.g., Nakayama, 1990; Van Essen, Olshausen, Anderson, & Gallant, 1991). It requires little effort to see relationships between switching tasks because of limited decision complexity and switching mode because of limited attention. In drawing this connection, one might say that if all vision results from performing a task, then in some sense one is always *attending*.

Treisman’s proposal of additional attentional modes seemed to resolve problems with earlier versions of selective attention theory. It paved the way to further studies on what information becomes available upon diffusely attending to a scene or a set (Alvarez, 2011; Fei-Fei, Iyer, Koch, & Perona, 2007; Greene & Oliva, 2009; Leib, Kosovicheva, & Whitney, 2016). However, this proposal also raises several questions that I will now address.

#### How dynamic is visual processing?

What, for instance, are the mechanisms associated with diffuse attention? How does the brain switch attentional modes, and how do upstream processes deal with changes in the encoding of available information? Different attentional modes suggest that from moment to moment the information encoded by the visual system can change dramatically with the focus and type of attention. To set up a classifier to perform a task, the visual system must know and adapt to the particular encoding that results from the current attentional state. Later processes must somehow deal with the highly dynamic nature of the encoded information.

Changing the task to accommodate limited decision complexity does not raise the same issues. Rather, each new task requires a late mechanism to set up a new classifier and interpret its results (though one may perhaps see effects of this mechanism early in visual processing as well). This theory presumes that, to a first approximation, changing the task changes neither the encoding nor the available information. Rather, each new query changes what we *know*. In our earlier example, the answer to the question of whether the pedestrians are near or far gives us new understanding of the scene.

#### What is the limit?

If diffuse attention and focal attention both satisfy a single capacity limit, then how should we conceptualize that capacity limit? In other words, in what sense might these two attentional modes be equivalent in terms of use of available resources? Understanding the answer would seem to be critical for characterizing, and thus predicting, how much detail is available under diffuse attention to a scene. Several researchers have speculated about the answer to this question (Franconeri, Alvarez, & Cavanagh, 2013; Nakayama, 1990; Van Essen, Olshausen, Anderson, & Gallant, 1991). Van Essen et al. (1991), for instance, suggested that the visual system might always have access to an approximately 25 × 25 array of feature vectors. These feature vectors could be spread either over an object or over the entire scene and might derive from any layer in the visual processing hierarchy. While this proposal is intriguing, it has not been obvious how to advance this theory.

On the other hand, while the exact nature of the decision complexity limit remains unclear, there would appear to be a viable path forward. We could use vision science’s considerable understanding of human behavioral limits to look for a consistent complexity limit such as those described above: number of hyperplanes, number of dimensions, curvature of the decision boundary, and so on. The limit might take other forms more specific to the physiology of the brain (VanRullen et al., 2004); if, for instance, the brain implemented classification tasks using center-surround mechanisms operating in some feature space, then the limit could instead be on the number or density of those mechanisms (Franconeri et al., 2013). Machine learning also has a concept of decision complexity and can provide other forms that this limit might take (e.g., Vapnik & Chervonenkis, 1971). Of course, looking for a consistent limit requires a model of the perceptual encoding space, but vision research has advanced to the point where one may feasibly use either computational models, such as trained CNNs, or rich, high-dimensional data from physiology, such as from fMRI. An understanding of possible decision limits, in turn, should make testable predictions of what tasks observers can and cannot do.

### A predictive, testable theory

The proposed theory—that peripheral encoding plus limited decision complexity explain the awareness puzzle and support real-world vision—has advantages over previous explanations simply in that it is predictive, and testable. Ask theories with pathways or modes for processing scenes to predict what scene tasks will be easy or hard, and researchers will run scene perception experiments to find out the answer. Ask what information the visual system encodes in a proto-object representation, and one can conduct experiments to find out. What detail is available? Run an experiment and find out. (Note, however, the potential for peripheral vision confounds in all of these experiments.) Though in some sense these theories provided a solution to the awareness puzzle and to how real-world vision works, they are essentially descriptive rather than predictive.

The proposed theory, on the other hand, has a concretely defined peripheral encoding. This specificity provides testable predictions about what details will be available at a glance, what search tasks will be easy or hard, and what scene and set tasks are possible, given the information that survives or is lost in peripheral vision. My lab has already demonstrated that this peripheral encoding predicts performance on a wide range of such tasks (Balas et al., 2009; Chang & Rosenholtz, 2016; Ehinger & Rosenholtz, 2016; Keshvari & Rosenholtz, 2016; Rosenholtz, Huang, & Ehinger, 2012; Rosenholtz, Huang, Raj, et al., 2012; Zhang et al., 2015).

Furthermore, the section “What Is the Limit” sketches a path toward fleshing out and testing the decision complexity part of the proposed theory. If there proves to be a consistent limit on decision complexity, this has additional implications. First, simplicity: This theory would unify understanding of different modes and types of attention via a single complexity limit, perhaps replacing a number of distinct limits and mechanisms. This may provide insight not only into vision per se, but also into visual working memory. Second, if correct, this theory should someday allow us to predict task difficulty based on a combination of peripheral factors and decision complexity.

### Revisiting illusion and inaccessibility theories

In the proposed theory, the perceptual encoding has more information than one can implicitly or explicitly understand at a given moment, because understanding only results from performing a task. Similarly, in Rensink’s (2000) theory, the proto-object representation contains more information than one can access at a given moment. Likely, all vision science theories have this kind of inaccessibility; there no doubt exist plenty of visual tasks humans perform poorly even though the retina has the necessary information.

However, the proposed theory is not an *inaccessibility theory*, in the sense that it does not use inaccessibility to explain the awareness puzzle. Rather, all tasks, including both traditional tasks and *awareness tasks*, encounter the same decision limits. Some tasks simply fare better under these limits than others. There is no need to postulate that awareness has access to information that is inaccessible to action and decision-making.

That perception results from inference suggests that there is some truth to the illusion theories of awareness. One perceives the results of inference, not some image captured by the eye-as-camera, and projected onto an internal screen for viewing by the homunculus. In this sense, perception is inherently something of an illusion. However, the illusion is not as extreme as previously thought, because vision is less impoverished than it would be if the classic theories about selective attention were correct. Thus, the rich percept is less surprising.

## Conclusions: A proposed explanation

**Fig. 12:**
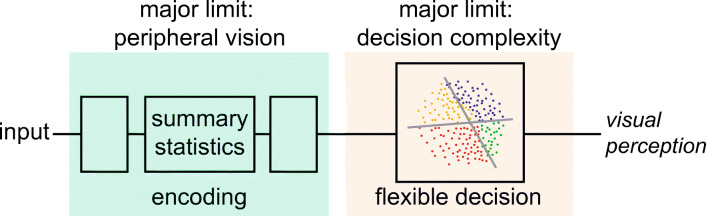
Proposed architecture. All visual perception arises from flexible decision mechanisms, operating on the perceptual encoding, to perform a task. The major limit on available information arises from a summary statistic encoding in peripheral vision, though other encoding losses occur as well. Decision mechanisms, while flexible, cannot implement arbitrarily complex decisions; a second major factor limiting performance of visual tasks.

I have argued that the strengths and limitations of visual perception result from constraints on both perceptual encoding and decision complexity. A visual task can be difficult because of either or both of these causes (see Fig. 12).

First, a striking number of puzzling visual phenomena can be explained by the information preserved and lost in peripheral vision. This paper discusses a concrete model of peripheral encoding; peripheral vision appears to encode its inputs in terms of a rich set of summary image statistics, computed by pooling image measurements across sizeable regions of the visual field. These regions grow—and the resulting summary statistics become increasingly less informative—with distance from the point of gaze. At a given moment, the current fixation largely determines the information available across the field of view. If a task needs information that does not survive the peripheral encoding, that task will be difficult. To gather more information, observers must move their eyes. This model has produced testable predictions showing that losses of peripheral information lead to poor performance on a number of visual tasks (difficult search, change blindness), while preserving sufficient information to make other tasks relatively easy (easy search, easy change detection, and getting the gist of a scene or set), and to support our rich percept of the world.

However, some tasks are difficult even if the necessary information survives both peripheral vision and the perceptual encoding stages more generally. I have argued that the second big piece of the solution has to do with decision limits, and in particular with limits on decision complexity. Dual tasks may be more difficult than single tasks because they are inherently more complex. Inattentional blindness—the inability to perform a task when it is unexpected—may occur when limits on decision complexity preclude performing both the nominal task and, by chance, also the unexpected task. MOT and VWM may both be inherently complex tasks, leading to apparent limits on the number of items that can be tracked or remembered.

Even if one thinks of the proposed decision complexity limit as a mere reframing of different attentional modes in terms of switching tasks to deal with limited decision complexity (the block diagrams certainly look similar; compare Fig. 6b with Fig. 12), the present hypothesis has a number of advantages. The proposed theory replaces multiple kinds of attention with a single complexity limit. It illuminates a path forward to understanding that limit. If successful, it could ultimately make testable predictions.

If attentional limits and mechanisms operated early in visual processing, then they would not obviously connect to other, presumably later, limits on visual working memory and cognition. However, if the limit is late, as is the case for decision complexity, this raises the possibility that that limit might be a general-purpose cognitive capacity limit. In fact, there is some evidence for this, from analysis of individual differences. Huang, Mo, and Li (2012) found correlated performance at a wide range of tasks, including search, counting, tracking, response selection, short-term memory, visual marking, task switching, and mental rotation.

Tasks that seem to show impoverished vision may simply be difficult tasks, either due to the encoding or due to limits on inference processes. On the other hand, perception is rich, and real-world vision successful, because the information for many tasks survives encoding losses, and that encoding evolved to make those tasks relatively simple. Importantly, to make sense of decision complexity, it helps to think about all visual perception as arising from performance of a visual task. This allows us to put all phenomena on the same footing; search, set perception, scene perception, visual working memory, multiple object tracking, Posner cueing, dual-task, change blindness, inattentional blindness, and visual awareness may encounter the same limits on both the information encoded and the complexity of decisions. Given those limits, some tasks may simply be inherently difficult, and others easy. If so, there is no need to ponder why, for instance, we get a rich subjective impression and yet do poorly at certain tasks; no need to postulate that the details are puzzlingly inaccessible for decision and action. If a unifying explanation is possible, there is no awareness puzzle.

## Footnotes

1 Note that while Rensink, O’Regan, and Clark (1997) interpret their results in terms of an illusion (my word, not theirs) of rich detail, supported by rapid switching of a low-capacity mechanism to provide details on demand, Rensink’s (2000) theory better fits into the second set of vision science theories, described later in this section.

2 Note that this encoding is not foveated. Despite the importance of peripheral vision for understanding many relevant perceptual phenomena, for this mini experiment we use an encoding that does not depend upon distance from the point of gaze.

3 Of course, one does not require such a mechanism to explain why observers perform better with a valid cue, as even a parallel, unlimited-capacity model predicts the existence of cueing effects. See, for example, Palmer, Ames, and Lindsey (1993).
